# Criterion Validity of Smartwatches for Measuring Heart Rate and Energy Expenditure During Resistance Exercise

**DOI:** 10.3390/bioengineering13101099

**Published:** 2026-09-23

**Authors:** Jung-Hyun Yun, Jae-Myun Ko, Tae-Hyung Lee

**Affiliations:** 1 Department of Sports Science, Konkuk University, Chungju 27478, Republic of Korea; chyun1221@kku.ac.kr; 2 Exercise Physiology Laboratory, Department of Physical Education, Yonsei University, Seoul 03722, Republic of Korea; kjm1218@yonsei.ac.kr

**Keywords:** criterion validity, energy expenditure, heart rate, photoplethysmography, resistance exercise, smartwatch, wearable sensors

## Abstract

Commercial smartwatches are increasingly used to monitor physiological responses during exercise; however, their validity for resistance exercise remains uncertain. This study evaluated the criterion validity of four commercial smartwatches (Apple Watch Series 8, Samsung Galaxy Watch 5, Fitbit Charge 5, and Garmin Vivosmart 5) for measuring heart rate (HR) and energy expenditure (EE) during resistance exercise. Seventy healthy men aged 20–39 years performed two sets each of the bench press, T-bar row, squat, and deadlift at individualized 10-repetition maximum loads. Smartwatch-derived HR was evaluated against electrocardiography, whereas smartwatch-derived workout-session EE was evaluated against indirect calorimetry. The Apple Watch showed the lowest measurement error for mean HR across the protocol (bias = −0.36 bpm; MAE = 1.81 bpm; ICC = 0.969) and for overall dynamic HR (bias = −0.72 bpm; MAE = 4.95 bpm; ICC = 0.904). HR agreement deteriorated markedly immediately after each set, particularly for the Samsung and Fitbit devices, which underestimated ECG-derived HR by 25.60 and 19.59 bpm, respectively. In contrast, EE estimates showed poor agreement with indirect calorimetry across all devices (ICC = 0.021–0.033). These findings demonstrate that the agreement of smartwatch-derived HR with electrocardiography measurements varies across time points during resistance exercise and indicate that smartwatch-derived workout-session EE should be interpreted cautiously when accurate metabolic assessment is required.

## 1. Introduction

Wearable technology has become one of the most rapidly expanding areas in exercise science and the fitness industry. Ranked as the leading fitness trend in both 2025 and 2026, wearable technology remains a major focus of interest among practitioners, clinicians, researchers, and consumers [1,2]. Wrist-worn devices such as smartwatches can continuously capture physiological and behavioral metrics, including heart rate (HR), physical activity, sleep, and energy expenditure (EE). Their applications have expanded from personal health tracking to exercise prescription, sports monitoring, and clinical rehabilitation [3,4,5,6,7].

Among the metrics tracked by smartwatches, HR is one of the most widely used indicators of exercise intensity, cardiovascular strain, and physiological responses to exercise [8]. Target HR, HR reserve, and post-exercise HR recovery are commonly used in exercise prescription and rehabilitation to guide exercise intensity, monitor recovery, and assess exercise safety [9,10]. However, before smartwatch-derived HR measurements can be used to inform exercise prescription or clinical decision-making, their agreement with criterion measures must be established [5,11].

Most commercial smartwatches estimate HR using photoplethysmography (PPG). Although PPG is convenient and enables continuous monitoring, its accuracy can be affected by sensor–skin contact, wrist movement, local blood flow, skin characteristics, exercise intensity, and activity type [11,12]. Commercial PPG systems may also differ in optical design, including light-emitting diode (LED) wavelength, emitter–detector geometry, the number of optical channels, and proprietary signal-processing algorithms, potentially contributing to device-specific differences in measurement performance [12]. During exercise, motion artifacts and changes in peripheral hemodynamics may degrade PPG signal quality; therefore, agreement established under resting conditions cannot necessarily be generalized to dynamic exercise. Accordingly, the INTERLIVE Network has recommended standardized exercise protocols, appropriate criterion measures, time-synchronized data processing, and activity-specific validation when evaluating HR measurements obtained from commercial wearable devices [11].

Previous studies have suggested that commercial wearable devices can provide acceptable HR estimates under controlled conditions, particularly at rest or during rhythmic aerobic exercise [4,13]. However, measurement accuracy may decrease during exercise and vary by activity type, particularly during activities involving substantial arm movement or motion artifacts [14,15]. Compared with HR measurements, smartwatch-derived EE estimates generally exhibit lower accuracy. Smartwatches estimate EE algorithmically using HR, movement data, user characteristics, GPS data, and other sensor inputs; however, these algorithms are typically proprietary and not publicly disclosed. Consequently, the accuracy of EE estimates may vary substantially according to the user, device, and activity performed [13,16,17].

Most studies have evaluated smartwatch validity at rest and during walking, running, cycling, or cardiopulmonary exercise. These activities typically involve rhythmic movement patterns and relatively stable cardiorespiratory responses. In contrast, resistance exercise is characterized by brief bouts of muscular contraction, intermittent recovery periods, substantial grip force and forearm muscle activation, wrist flexion and extension, postural changes, and alterations in local blood flow. Mechanical loading associated with gripping and forearm muscle contraction may alter sensor–skin pressure and induce wrist movement, thereby creating additional sources of PPG artifacts during resistance exercise [12,18]. Consistent with this mechanism, Jo et al. [19] reported reduced accuracy of wrist PPG-derived HR during exercise tasks involving sustained or repetitive forearm muscle contractions and proposed that forearm muscle activity and sensor–skin compression may interfere with optical signal acquisition. HR and oxygen uptake may also remain in a non-steady state and change rapidly during transitions between work and recovery phases. Collectively, these physiological and mechanical characteristics may compromise wrist PPG signal acquisition and challenge the algorithms that integrate HR and accelerometry to estimate metabolic demand.

Studies evaluating smartwatch validity during resistance exercise remain limited. Boudreaux et al. [18] reported that HR validity varied across devices and exercise conditions and that EE was inaccurately estimated during cycling and resistance exercise. More recently, our previous study published in Sensors [6] compared Apple, Galaxy, Fitbit, and Garmin devices during endurance and resistance exercise and identified exercise modality-dependent differences, with resistance exercise posing greater measurement challenges. The present study was designed as an independent follow-up investigation motivated by these findings. It included a separate participant cohort, followed a separate IRB-approved protocol, used different Apple and Samsung hardware and software configurations, and included no participant-level data from the previous study. Whereas the previous study primarily compared validity across exercise modalities, the present study focuses specifically on resistance exercise and evaluates HR agreement at set-related time points and across exercise contexts. Thus, it examines whether smartwatch-displayed HR differs from electrocardiography (ECG)-derived HR during the rapid physiological transition immediately after each resistance exercise set, rather than treating resistance exercise as a single averaged condition.

Therefore, this study evaluated the criterion validity of four commercial smartwatch models for measuring dynamic HR and EE during resistance exercise in healthy young men. Specifically, HR measured by the Apple Watch Series 8, Galaxy Watch 5, Fitbit Charge 5, and Garmin Vivosmart 5 was compared with ECG-derived HR during the bench press, T-bar row, squat, and deadlift. In addition to the pre-protocol laboratory baseline and mean HR across the protocol, we assessed HR immediately before each set, immediately after each set, and 1 min after each set to determine how agreement varied across rapid physiological transitions. Manufacturer-reported workout-session EE was compared with EE derived from indirect calorimetry over the corresponding protocol interval. By characterizing device-, time point-, and exercise-specific error patterns, this study sought to provide practical guidance for interpreting smartwatch-derived HR and EE measurements during resistance exercise.

## 2. Materials and Methods

### 2.1. Design and Participants

This study used a repeated-measures experimental design to evaluate the criterion validity of commercial smartwatches for measuring HR and EE during resistance exercise. Participants were recruited through convenience sampling from the university and local community using institutional review board-approved recruitment materials, including campus flyers and online advertisements. The recruitment materials briefly described the study purpose, eligibility criteria, experimental procedures, potential risks, and research team contact information. Individuals interested in participating contacted the research team or completed an initial screening form. The investigators then confirmed eligibility before enrollment. The inclusion criteria were as follows: healthy, non-athlete adult men; prior resistance-exercise experience sufficient to safely perform the prescribed bench press, T-bar row, squat, and deadlift; no restrictions that would preclude participation in resistance exercise; and provision of written informed consent. The duration and frequency of prior resistance-training experience were not systematically quantified. For this study, “healthy” was operationally defined as apparently healthy and able to safely perform the required resistance exercises based on a preparticipation screening process informed by the recommendations of the American College of Sports Medicine [10,20]. Specifically, eligible participants were required to report no known cardiovascular, metabolic, renal, neurological, or musculoskeletal diseases; no signs or symptoms suggestive of cardiovascular disease; no current use of medications that could affect HR responses; and no pain, injury, or functional limitations that would restrict their ability to perform the bench press, T-bar row, squat, or deadlift. Participants were also instructed to refrain from high-intensity exercise and from consuming alcohol or excessive amounts of caffeine for 24 h before testing.

A total of 70 healthy, non-athlete men aged 20–39 years participated in the study. The participants had a mean age of 26.49 ± 4.02 years, height of 175.09 ± 4.61 cm, body mass of 73.54 ± 7.50 kg, body mass index of 23.98 ± 2.12 kg/m^2^, body fat mass of 13.17 ± 4.18 kg, and skeletal muscle mass of 34.32 ± 3.29 kg. All participants provided written informed consent. The study protocol was approved by the Institutional Review Board of the Korea National Institute for Bioethics Policy (IRB No. P01-202606-01-098; approval date: 19 June 2026), and all procedures were conducted in accordance with the principles of the latest revision of the Declaration of Helsinki.

### 2.2. Measurement of Anthropometry and Body Composition

Participants’ height and body mass were measured using an automated stadiometer and scale (BSM330; Biospace, Seoul, Republic of Korea). Body composition was assessed using bioelectrical impedance analysis (InBody720; Biospace, Seoul, Republic of Korea), and body fat mass and skeletal muscle mass were recorded. All measurements were obtained under standardized pre-exercise conditions, and participants were instructed to remove all metal objects before measurement.

### 2.3. Measurement of ECG-Derived HR and Indirect Calorimetry-Derived EE

HR was measured using a standard ECG exercise-testing system (Quinton Q-Stress System, version 4.5; Cardiac Science Corporation, Bothell, WA, USA) with disposable ECG electrodes (Kendall; Cardinal Health, Dublin, OH, USA). ECG signals were acquired continuously throughout the resting, exercise, and recovery periods. At each predefined observation time point, the HR value displayed in real time on the Q-Stress monitor was recorded. According to the manufacturer’s signal-analysis documentation, the displayed HR value is calculated from the preceding 6 s of ECG data and updated every 2 s. EE was determined by indirect calorimetry using a metabolic measurement system (TrueOne 2400; ParvoMedics, Sandy, UT, USA), which served as the criterion method for EE assessment. Oxygen consumption and carbon dioxide production were measured continuously throughout the exercise-session recording interval, and EE was calculated from the measured respiratory gas exchange using the abbreviated Weir equation, as described in Section 2.6. The metabolic measurement system was calibrated for flow and gas concentrations before each test in accordance with the manufacturer’s instructions.

### 2.4. Smartwatches and Placement Randomization

Four commercial smartwatches were evaluated: Apple Watch Series 8 (Apple Inc., Cupertino, CA, USA), Samsung Galaxy Watch 5 (Samsung Electronics, Seoul, Republic of Korea), Fitbit Charge 5 (Fitbit Inc., San Francisco, CA, USA), and Garmin Vivosmart 5 (Garmin Ltd., Schaffhausen, Switzerland). Sex, age, height, and body mass were entered into each device using standardized procedures. Device and companion-app configurations were kept consistent throughout data collection. The Apple Watch Series 8 used watchOS 26.5 and was paired with an iPhone running iOS 26.5 using the native Fitness application; the Traditional Strength Training mode was used for all four exercises. The Galaxy Watch 5 used firmware DZB6 with Galaxy Wearable version 2.2.68.26010761 and Samsung Health version 7.00.0.107; the exercise profiles used were Bench press, Weight machine for the T-bar row, Squats, and Deadlifts. The Fitbit Charge 5 used firmware 194.91 with Fitbit app version 5.01.1 and the Weights mode for all exercises. The Garmin Vivosmart 5 used software version 3.32 with Garmin Connect version 5.26 and the Strength Training mode for all exercises. Workout recording was initiated manually on each device; no additional investigator-applied HR smoothing or post-processing was performed. Manufacturer-implemented filtering, smoothing, and display-refresh algorithms were proprietary and could not be independently configured by the investigators.

Because device placement may influence PPG measurement accuracy, the four smartwatches were randomly assigned, for each participant, to one of four positions: the proximal left wrist, distal left wrist, proximal right wrist, or distal right wrist. Device positions were varied across participants to minimize systematic manufacturer-specific placement bias. Stable sensor–skin contact was confirmed, and the devices were positioned to prevent overlap. However, participant-level device-allocation records were not retained; therefore, the frequency with which each model was assigned to each wrist position could not be reconstructed, and wrist side and proximal/distal position could not be included as factors in the statistical analyses.

### 2.5. Resistance-Exercise Protocol

All participants completed a standardized resistance-exercise protocol in the same laboratory environment. The protocol consisted of four resistance exercises, each performed for two sets at an individualized 10-repetition maximum (10RM) load. The exercises were performed in the following fixed order: bench press, T-bar row, squat, and deadlift; this sequence was repeated for the second set. A 3 min seated recovery period was provided after each exercise bout throughout the protocol, thereby providing a standardized recovery interval before the subsequent bout, although complete physiological recovery was not assumed.

Each exercise was performed for 10 repetitions at a metronome-controlled tempo of 2 s for the concentric phase and 2 s for the eccentric phase; thus, each exercise bout lasted approximately 40 s. All participants completed the prescribed repetitions in both sets while maintaining the metronome-paced cadence, and no repetition or set was terminated because of task failure. The standardized resistance-exercise protocol lasted approximately 34 min and included eight 40 s exercise bouts, eight 3 min seated recovery periods, and brief transition and measurement intervals. The 3 min pre-protocol laboratory baseline was obtained before the exercise-session recording interval and was excluded from EE accumulation. The final 3 min recovery period after the last exercise bout was included in the common exercise-session endpoint used to temporally align HR and EE measurements. All exercises were supervised by trained staff, who provided verbal instructions to ensure proper exercise technique and adherence to the prescribed cadence.

Before the standardized baseline measurement and resistance-exercise protocol, participants completed a supervised warm-up and underwent 10RM determination for each exercise. The 10RM load was defined as the maximum load that could be lifted for 10 repetitions with correct technique. If an attempt was unsuccessful or exercise technique deteriorated, the load was adjusted after a sufficient rest period, and the final successfully completed 10RM load for each participant was used in the protocol. Participants then completed a seated recovery period before the 3 min pre-protocol baseline measurement. The same individualized 10RM load was used for both sets of each exercise.

### 2.6. HR and EE Data Processing

A pre-protocol laboratory baseline, mean protocol HR, and time point-specific HR measurements were evaluated. After 10RM determination and seated recovery, participants sat quietly for 3 min; the HR value displayed at the end of this period was used as the pre-protocol laboratory baseline. Because this measurement was obtained after 10RM testing and subsequent recovery, it was not interpreted as habitual resting HR. Mean protocol HR was defined as the mean HR over the standardized resistance-exercise recording interval. For each resistance exercise set, HR was recorded immediately before the set, immediately after the set, and 1 min after the set. Immediate post-set HR was defined as the value visible on the device display within 2 s after completion of the 10th repetition. Three trained investigators jointly monitored the ECG and smartwatch displays and manually recorded the HR values displayed simultaneously at the predefined event-based observation time points. Thus, smartwatch HR was obtained directly from the real-time device displays rather than from HR data subsequently exported from the companion applications. Completion of the final repetition served as the common temporal reference point; retrospective timestamp matching was not performed. No scheduled HR measurement was missing because of a blank or delayed smartwatch display. Device-specific display-refresh intervals and proprietary averaging windows were not available to the investigators. HR error was calculated as smartwatch-displayed HR minus ECG-displayed HR; positive values indicated overestimation by the smartwatch, whereas negative values indicated underestimation. For EE assessment, after the pre-protocol baseline measurement and completion of all measurement setup procedures, smartwatch workout recording and indirect calorimetry were initiated simultaneously at the start of the resistance-exercise protocol and terminated simultaneously at the predefined protocol endpoint. Recording continued throughout all exercise bouts, scheduled recovery periods, and transition intervals within the exercise session. Immediately after completion of the protocol, the single workout-session EE value (kcal) displayed in each manufacturer’s companion application for the completed resistance-training session was visually confirmed and manually recorded; daily total calorie values were not used. Criterion EE was derived from oxygen consumption (VO_2_) and carbon dioxide production (VCO_2_) measured continuously using the TrueOne 2400 over the identical recording interval and was calculated using the abbreviated Weir equation [21]: EE (kcal·min^−1^) = 3.941 × VO_2_ (L·min^−1^) + 1.106 × VCO_2_ (L·min^−1^). EE error was calculated as smartwatch-derived EE minus indirect calorimetry-derived EE.

### 2.7. Statistical Analysis

Data are presented as means and standard deviations. Normality was assessed using the Shapiro–Wilk test and Q–Q plots. Statistical significance was set at α = 0.05. Statistical analyses were performed using R version 4.3.3 (R Foundation for Statistical Computing, Vienna, Austria). Descriptive results were cross-checked, when necessary, using IBM SPSS Statistics version 31.0 (IBM Corp., Armonk, NY, USA).

Measurement accuracy was quantified using mean bias, mean absolute error (MAE), mean absolute percentage error (MAPE), and root mean square error (RMSE). Absolute agreement was assessed using intraclass correlation coefficients (ICCs) derived from a two-way mixed-effects, absolute-agreement, single-measure model [22]. ICCs were interpreted as continuous measures of agreement rather than categorized according to predefined thresholds of practical acceptability. Mean bias and ICCs are reported with 95% confidence intervals (CIs), estimated using participant-clustered bootstrap resampling with 2000 resamples. For the overall dynamic-HR analysis, the primary ICC analysis treated each measurement occasion as the paired observational unit, while preserving repeated observations within participants during bootstrap resampling. To assess the robustness of the ICC results to the choice of statistical target, a sensitivity analysis was conducted in which the 24 dynamic-HR observations were first averaged for each participant and device. The absolute-agreement, single-measure ICC was then recalculated using the 70 participant-level paired means, with 95% CIs again estimated by participant-level bootstrap resampling.

Agreement between smartwatch-derived and criterion measurements was evaluated using Bland–Altman analysis, with mean bias and 95% limits of agreement (LoA) reported. For dynamic HR analyses involving multiple observations per participant, repeated-measures Bland–Altman methods were used to account for within-participant dependence [23]. To determine whether HR error differed according to device, exercise, time point, set, or prespecified interactions, repeated-measures fixed-effects models were fitted with HR error (smartwatch-displayed HR minus ECG-displayed HR) as the dependent variable. Prespecified fixed effects included device, exercise, time point, set, device × time point, and device × exercise. To evaluate the hypothesized variation in temporal error patterns across exercises, a sensitivity model additionally included the exercise × time point interaction. A full device × exercise × time point interaction was not included because it was not prespecified, the fixed exercise sequence precluded separation of exercise effects from protocol-position effects, and additional three-way parameterization would not resolve this design-related confounding. Participant-clustered robust standard errors were used to account for repeated observations within participants. A random-intercept mixed-effects model was initially considered; however, the estimated random-intercept variance approached zero, resulting in a singular fit. Therefore, final inference was based on the fixed-effects model with participant-clustered robust standard errors. Participant-clustered bootstrap resampling was also used to estimate marginal mean HR errors and corresponding 95% CIs by device, time point, exercise, and set. Pairwise post hoc comparisons of participant-level mean HR error between devices were performed for the overall, time point-specific, and exercise-specific analyses using paired t tests when the paired differences were normally distributed and Wilcoxon signed-rank tests otherwise, with Holm adjustment applied to account for multiple comparisons. Detailed sensitivity-analysis and post hoc results are provided in Supplementary Tables S1–S6.

## 3. Results

### 3.1. Validity of Pre-Protocol Laboratory Baseline HR, Mean Protocol HR, and EE

The criterion-validity results for smartwatch-derived pre-protocol laboratory baseline HR, mean protocol HR, and EE are presented in Table 1 and Figure 1. For the pre-protocol laboratory baseline, the Apple Watch Series 8 showed the smallest absolute error and highest ICC (bias = −0.90 bpm [95% CI: −1.89 to 0.04], MAE = 3.16 bpm, ICC = 0.963 [95% CI: 0.937 to 0.978]), whereas the Galaxy Watch 5 showed wider LoA despite a small mean bias. For mean protocol HR, the Apple Watch Series 8 again showed the smallest error and highest ICC (bias = −0.36 bpm [95% CI: −1.16 to 0.30], MAE = 1.81 bpm, ICC = 0.969 [95% CI: 0.929 to 0.989]). The Galaxy Watch 5, Fitbit Charge 5, and Garmin Vivosmart 5 showed mean biases of −4.36, −3.40, and −2.44 bpm, respectively, indicating underestimation of mean protocol HR. In contrast, manufacturer-reported workout-session EE showed very low ICCs (0.021–0.033) and wide LoA relative to indirect calorimetry-derived EE. The Apple Watch Series 8, Galaxy Watch 5, and Garmin Vivosmart 5 substantially overestimated workout-session EE relative to indirect calorimetry, whereas the Fitbit Charge 5 showed a smaller mean bias but a large MAE and wide LoA (Figure 2). 

### 3.2. Overall Validity of Dynamic HR Measurements During Resistance Exercise

Table 2 presents the overall dynamic-HR results across four exercises, two sets, and three time points. The Apple Watch Series 8 showed the smallest error and highest ICC (bias = −0.72 bpm [95% CI: −1.19 to −0.24], MAE = 4.95 bpm, ICC = 0.904 [95% CI: 0.883 to 0.923]), followed by the Garmin Vivosmart 5 (bias = −3.95 bpm [95% CI: −4.50 to −3.37], MAE = 7.18 bpm, ICC = 0.796 [95% CI: 0.769 to 0.821]). The Galaxy Watch 5 and Fitbit Charge 5 showed larger negative mean biases, indicating greater underestimation of ECG-derived HR. When each participant’s 24 dynamic-HR observations were first averaged such that the participant served as the statistical target, the sensitivity-analysis ICCs were 0.963 (95% CI: 0.939–0.976) for the Apple Watch Series 8, 0.599 (0.506–0.667) for the Galaxy Watch 5, 0.713 (0.628–0.773) for the Fitbit Charge 5, and 0.841 (0.780–0.884) for the Garmin Vivosmart 5, with the relative ordering of the device-specific ICCs remaining unchanged (Supplementary Table S6).

### 3.3. Time Point-Specific Validity of Dynamic HR Measurements

The time point-specific results are presented in Table 3 and Figure 3 and Figure 4. Pre-set and 1 min post-set measurements showed relatively small mean biases and higher ICCs across devices. In contrast, measurement error increased and agreement decreased immediately after each set. The Galaxy Watch 5 and Fitbit Charge 5 showed pronounced underestimation of ECG-derived HR (bias = −25.60 bpm [95% CI: −27.20 to −24.01] and −19.59 bpm [95% CI: −20.98 to −18.17], respectively), whereas the Apple Watch Series 8 showed a smaller negative mean bias but a higher MAE (bias = −4.33 bpm [95% CI: −5.64 to −3.06], MAE = 9.59 bpm, ICC = 0.724 [95% CI: 0.651 to 0.785]). These results characterize disagreement between the simultaneously observed user-facing ECG and smartwatch displays at the predefined post-set observation time point; however, they do not distinguish raw sensor measurement error from manufacturer-specific signal-processing and display-update latency.

### 3.4. Exercise-Specific Validity of Dynamic HR Measurements

Table 4 and Figure 5 present the exercise-specific dynamic-HR results. The Apple Watch Series 8 showed comparatively consistent measurement errors across the four exercises, whereas the Galaxy Watch 5 and Fitbit Charge 5 showed larger errors during the squat and deadlift. For example, during the squat, the MAEs for the Galaxy Watch 5 and Fitbit Charge 5 were 13.67 and 13.13 bpm, respectively; during the deadlift, the corresponding values were 13.12 and 10.83 bpm. Because all participants completed the exercises in the fixed sequence of bench press → T-bar row → squat → deadlift, the observed exercise-specific differences were completely confounded with protocol position and accumulated prior workload and therefore cannot be causally attributed to the specific exercise performed.

### 3.5. Repeated-Measures Models

The repeated-measures model results are presented in Table 5. Dynamic HR error differed significantly by device, exercise/protocol stage, and time point (all *p* < 0.001), whereas the main effect of set was not significant (*p* = 0.985). The device × time point and device × exercise interactions were also significant (both *p* < 0.001). In an additional sensitivity analysis, the exercise × time point interaction was significant (Wald χ^2^(6) = 252.54, *p* < 0.001), indicating that the magnitude of HR error varied across time points depending on the fixed exercise/protocol stage. Across devices, the participant-clustered marginal mean HR error was 0.48 bpm (95% CI: 0.11 to 0.86) pre-set, −15.33 bpm (95% CI: −16.34 to −14.23) immediately post-set, and 0.53 bpm (95% CI: 0.19 to 0.87) at 1 min post-set. The marginal mean error was −4.77 bpm in both Set 1 (95% CI: −5.25 to −4.31) and Set 2 (95% CI: −5.17 to −4.36), consistent with the absence of a statistically significant main effect of set. Device-specific set estimates and additional marginal means are provided in Supplementary Tables S4 and S5. Holm-adjusted pairwise device comparisons are provided in Supplementary Tables S1–S3. EE error also differed significantly by device (*p* < 0.001).

## 4. Discussion

This study evaluated the criterion validity of four specific commercial smartwatch models for measuring HR and EE during resistance exercise in healthy young men. The central finding was that disagreement between smartwatch-derived and ECG-derived HR depended strongly on the measurement phase. Pre-protocol laboratory baseline HR and mean protocol HR showed relatively small measurement errors for some models, whereas error increased markedly immediately after each set and decreased again by 1 min post-set. The Galaxy Watch 5 and Fitbit Charge 5 showed particularly large negative mean biases immediately post-set, indicating substantial underestimation relative to ECG. Thus, the present results support distinguishing agreement for averaged or session-level HR from agreement during rapid post-set transitions rather than drawing a categorical conclusion that a smartwatch is globally accurate or inaccurate.

Mean protocol HR is a time-averaged quantity; therefore, transient signal loss, delayed display updating, or brief underestimation may have a reduced influence when HR values are averaged across the full session. Accordingly, a high ICC or small mean bias for mean protocol HR does not establish equivalent measurement performance during rapid physiological transitions. Recent reviews have emphasized that wearable HR performance varies by device, activity, and measurement context [7,24]. The present results further indicate that the timing of measurement within a resistance-exercise bout is an important determinant of the magnitude of disagreement. Because no a priori threshold for practical acceptability was specified, the results are interpreted in terms of error magnitude and agreement rather than using categorical labels such as acceptable, accurate, or useful.

The marked disagreement between smartwatch-displayed and ECG-displayed HR immediately post-set may reflect a combination of PPG measurement limitations, device-level signal processing, display-update behavior, and the physiological characteristics of resistance exercise. Wrist-worn PPG is sensitive to motion, sensor–skin contact, tissue optical properties, and peripheral hemodynamics [12]. Resistance exercise additionally involves substantial grip force and forearm muscle activation, changes in sensor–skin pressure, wrist movement, and rapid changes in vascular tone and local blood flow. Task-specific reductions in wrist PPG accuracy have likewise been reported during resisted arm raises, lunges, and isometric planks, for which sustained or repetitive forearm contractions were proposed as a potential contributor to optical signal degradation [19]. Collectively, these factors may plausibly degrade PPG signal quality during resistance exercise. However, the present study recorded user-facing display values and did not acquire raw PPG signals or device-internal timestamps; therefore, the observed post-set disagreement cannot be partitioned into raw sensor measurement error versus system-level signal processing or display-update latency.

The immediate post-set observation time point is physiologically relevant because cardiovascular responses change rapidly during early recovery. The Q-Stress criterion display itself represents HR calculated from the preceding 6 s of ECG data and updated every 2 s, whereas the proprietary averaging windows and display-refresh characteristics of the smartwatches were not available to the investigators. Consequently, the present study should not be interpreted as directly measuring sensor latency. Rather, it demonstrates that the user-facing smartwatch HR values available within 2 s after set completion showed substantially greater disagreement with the simultaneously observed ECG criterion display than did pre-set or 1 min post-set values. This distinction is important for applications in which users or practitioners read smartwatch-displayed HR immediately after a set.

Larger measurement errors were observed during the later squat and deadlift stages, particularly for the Galaxy Watch 5 and Fitbit Charge 5. Although the mechanical characteristics of these exercises—such as whole-body movement, grip, trunk stabilization, and forearm tension—could plausibly contribute to PPG artifacts, the present design does not permit these potential effects to be isolated from sequence-related factors. Exercise type was completely confounded with protocol position because every participant completed the bench press, T-bar row, squat, and deadlift in the same order in both sets. The significant exercise × time point interaction in the sensitivity analysis therefore indicates that the temporal pattern of HR error varied across the fixed exercise/protocol stages; however, the larger errors during the later stages may also reflect accumulated prior workload, fatigue, sweating, elapsed time, or other sequence-related factors. Future validation studies should randomize or counterbalance exercise order when the aim is to isolate exercise-specific effects on measurement performance.

This independent follow-up study extends our earlier findings published in *Sensors* [6], which identified exercise modality-dependent differences in smartwatch validity and motivated a more focused evaluation of resistance exercise. The present study used a separate participant cohort, a separate IRB-approved protocol, and different Apple and Samsung hardware and software configurations, with no shared participant-level data. Its additional contribution is the evaluation of agreement at pre-set, immediate post-set, and 1 min post-set time points and across fixed exercise/protocol stages. These findings indicate that resistance exercise should not be treated as a single homogeneous validation condition; however, stage-specific patterns must be interpreted in light of the fixed-order limitation.

Device-specific differences were also observed among the tested models. The Apple Watch Series 8 generally showed smaller measurement errors and higher agreement estimates than the Galaxy Watch 5, Fitbit Charge 5, and Garmin Vivosmart 5 under several conditions. These differences may reflect manufacturer-specific optical hardware, LED configurations, sensor geometry, signal-processing algorithms, motion-artifact reduction, smoothing and filtering procedures, and display-update behavior. Because the internal algorithms are proprietary, the present data cannot identify the mechanisms responsible for the observed model-specific error patterns. The findings therefore apply to the specific hardware and software or firmware configurations tested and should not be generalized to all products from a given manufacturer or to future software or firmware versions.

The EE findings provide a distinct but equally important message. The single workout-session calorie value reported in each manufacturer’s companion application for the completed resistance-training session showed poor agreement with EE derived from indirect calorimetry despite identical exercise-session start and end points. Commercial smartwatches estimate EE using proprietary algorithms that may integrate HR, movement, user characteristics, and exercise mode, whereas criterion EE in the present study was calculated from measured respiratory gas exchange. Because the manufacturers’ algorithms are not publicly disclosed, the specific sources of EE estimation error cannot be determined from the present data.

Nevertheless, the present pattern is consistent with previous validation research. Systematic reviews have repeatedly reported lower validity for wearable-derived EE than for HR, and substantial disagreement with indirect calorimetry has been observed across consumer devices and exercise conditions [4,13,17,24]. Boudreaux et al. [18] likewise reported limited EE validity during cycling and resistance exercise, and our previous independent study published in Sensors [6] also showed poor agreement between smartwatch-derived EE and indirect calorimetry during resistance exercise. Thus, the substantial disagreement observed in the present study is consistent with the broader validation literature rather than being unique to the devices and protocol evaluated here. When precise metabolic assessment is required, manufacturer-reported smartwatch EE estimates should therefore be interpreted cautiously.

Accurate EE estimation is particularly challenging during resistance exercise because the relationships among HR, accelerometry-derived movement signals, oxygen uptake, and metabolic cost are neither stable nor linear. Resistance exercise consists of brief, high-intensity muscular contractions interspersed with recovery periods. During exercise sets, substantial muscular tension, transient blood pressure responses, altered breathing patterns, Valsalva-like maneuvers, and increased intramuscular pressure may occur. After completion of a set, HR and blood pressure begin to recover; however, oxygen uptake and other aspects of metabolic recovery may follow different temporal patterns. These rapidly changing physiological relationships may limit the accuracy of generalized algorithms that estimate EE from HR and accelerometer-derived movement data. Together with previous evidence of limited EE validity during resistance exercise [18], the present findings support cautious interpretation of smartwatch-derived EE estimates during non-steady-state resistance exercise.

In this study, each smartwatch workout recording was manually started and stopped at the same time points as indirect calorimetry, and both measurement methods covered the same exercise-session interval, including scheduled recovery periods and transitions. The INTERLIVE Network recommends standardizing criterion measures, activity protocols, metric definitions, data processing, and reporting when validating wearable-derived EE [16]. The present procedure therefore provides a temporally aligned session-level comparison between manufacturer-reported workout calories and criterion EE calculated from measured respiratory gas exchange. However, because the internal EE algorithms are proprietary, the contributions of individual algorithmic components to the observed estimation error cannot be isolated.

From a practical perspective, the tested devices showed smaller measurement errors for mean protocol HR and 1 min post-set HR than for immediate post-set HR; however, the practical acceptability of a given error magnitude depends on the intended application. The present study did not prespecify a universal threshold for clinical or exercise-prescription acceptability. Immediate post-set values, particularly for models showing systematic underestimation, therefore warrant greater caution when users or practitioners require an HR value during the first seconds of recovery. Manufacturer-reported workout-session EE should likewise be interpreted as an algorithmically derived session-level estimate rather than as a precise measure of metabolic cost.

This study has several limitations. First, the sample consisted only of healthy men aged 20–39 years; equivalent measurement performance cannot be assumed in women, older adults, individuals with cardiovascular or metabolic disease, or rehabilitation populations. Second, prior resistance-training experience was sufficient for safe protocol completion but was not quantitatively recorded; therefore, potential effects of training history or movement proficiency could not be examined. Third, only the Apple Watch Series 8, Galaxy Watch 5, Fitbit Charge 5, and Garmin Vivosmart 5 with the reported software and firmware configurations were tested; therefore, the results should not be generalized to other models or future software or firmware updates. Fourth, device placement was randomized, but participant-level allocation records were not retained; therefore, the distribution of devices across wrist positions could not be reconstructed, and placement effects could not be analyzed. Fifth, skin pigmentation or phototype was not formally assessed. All participants were Korean men, and prior Korean population studies have reported Fitzpatrick type III as the most prevalent phototype (48.8–55.0%), followed by types IV and V [25,26], indicating that types III–V predominate at the population level. However, population-level distributions cannot be used to infer individual phototype, and phototype-specific PPG measurement performance could not be evaluated in the present cohort. Sixth, exercise order was fixed; therefore, exercise type was completely confounded with protocol position and accumulated prior workload. Seventh, raw PPG signals, device-internal timestamps, proprietary display-refresh and averaging intervals, wrist acceleration, sensor–skin contact pressure, skin blood flow, and blood pressure were not measured. Thus, interpretations regarding motion artifacts, grip and forearm effects, and system-level temporal behavior remain plausible explanations rather than mechanisms directly evaluated in this study. Finally, manufacturer-specific EE algorithms are proprietary; therefore, the specific sources of EE estimation error could not be determined.

## 5. Conclusions

This study evaluated the criterion validity of four specific smartwatch models during a standardized resistance-exercise protocol. Mean protocol HR and 1 min post-set HR generally showed smaller measurement errors and greater agreement with ECG than HR observed within 2 s after set completion, with the magnitude of immediate post-set disagreement varying by device and fixed exercise/protocol stage. Because exercise order was fixed, the larger measurement errors observed during the later squat and deadlift stages cannot be separated from effects related to protocol position or accumulated prior workload. Manufacturer-reported workout-session EE estimates showed poor agreement with EE derived from indirect calorimetry despite identical recording intervals, supporting cautious interpretation of these estimates when precise metabolic assessment is required. These findings are specific to the tested models and software and firmware configurations and support time point- and model-specific interpretation of smartwatch-derived HR measurements during resistance exercise.

## Figures and Tables

**Figure 1:**
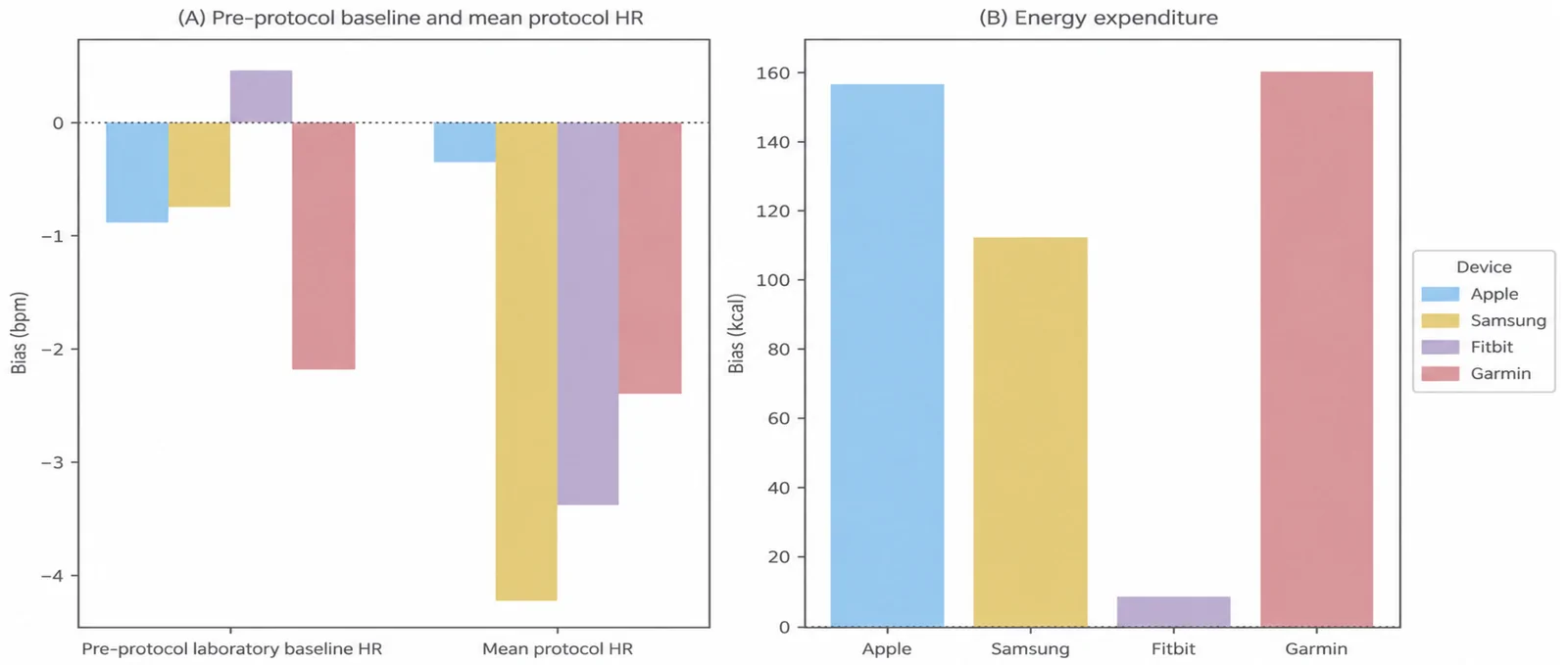
Mean bias of smartwatch-derived pre-protocol laboratory baseline HR, mean protocol HR, and energy expenditure. (**A**) Bias in pre-protocol laboratory baseline HR and mean protocol HR, calculated as smartwatch-derived HR minus ECG-derived HR. (**B**) Bias in smartwatch-derived EE, calculated as smartwatch-derived EE minus indirect calorimetry-derived EE. Negative HR bias values indicate underestimation relative to ECG, whereas positive EE bias values indicate overestimation relative to indirect calorimetry.

**Figure 2:**
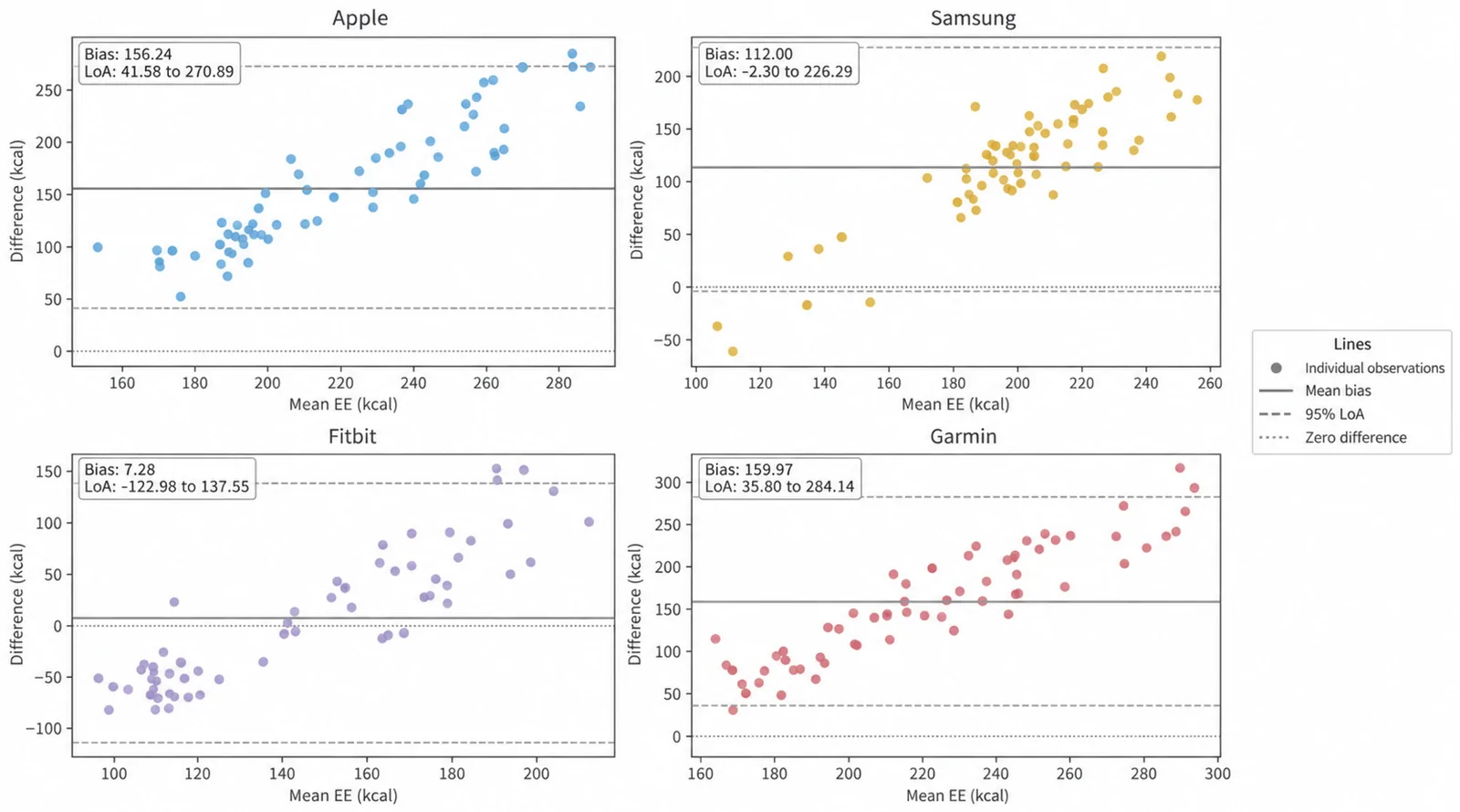
Bland–Altman plots of smartwatch-derived energy expenditure. Each panel represents one smartwatch. The difference was calculated as smartwatch-derived energy expenditure minus indirect calorimetry-derived energy expenditure. Positive values indicate overestimation relative to indirect calorimetry.

**Figure 3:**
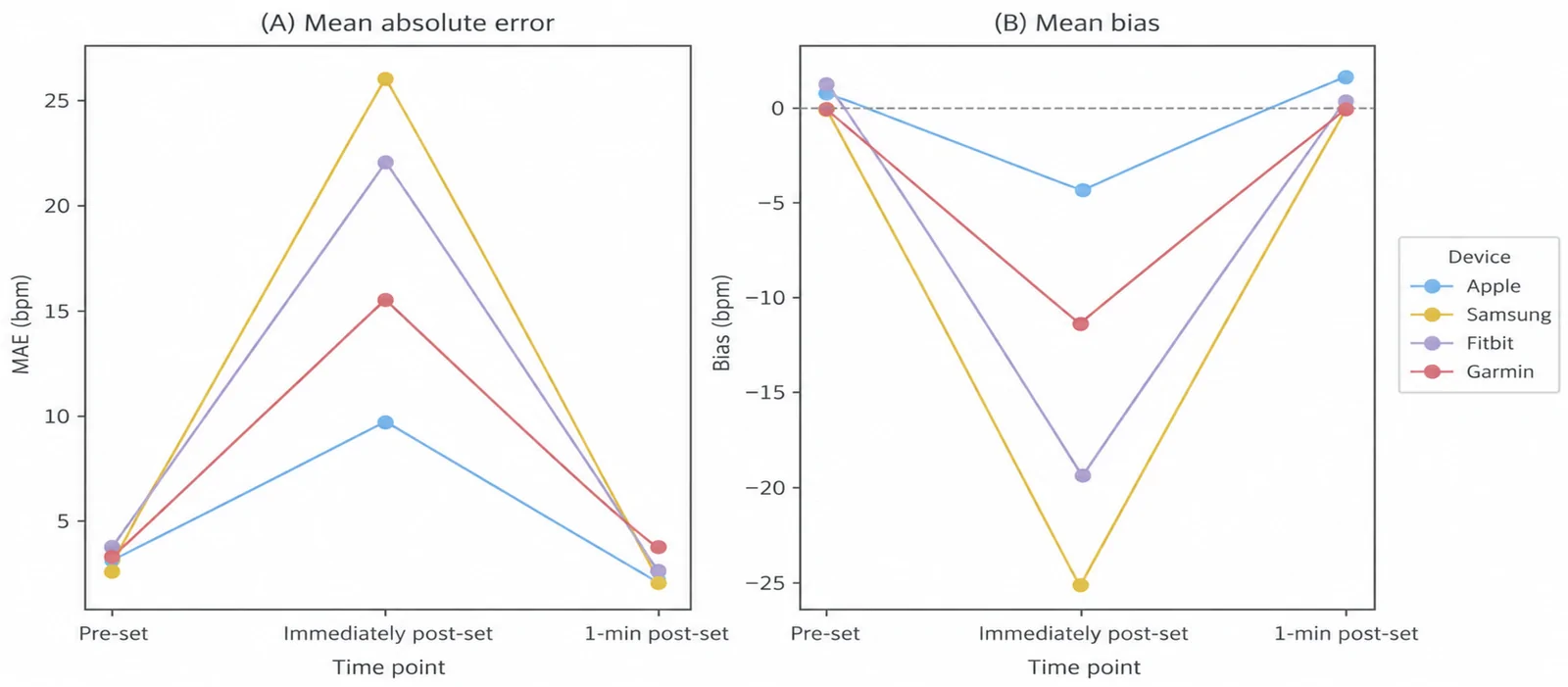
Time point-specific errors in smartwatch-derived heart rate during resistance exercise. (**A**) Mean absolute error of smartwatch-derived heart rate across time points. (**B**) Mean bias in smartwatch-derived heart rate across time points. Bias was calculated as smartwatch-derived heart rate minus ECG-derived heart rate. Positive values indicate overestimation relative to ECG, whereas negative values indicate underestimation. Measurement error was greatest immediately post-set. MAE = mean absolute error.

**Figure 4:**
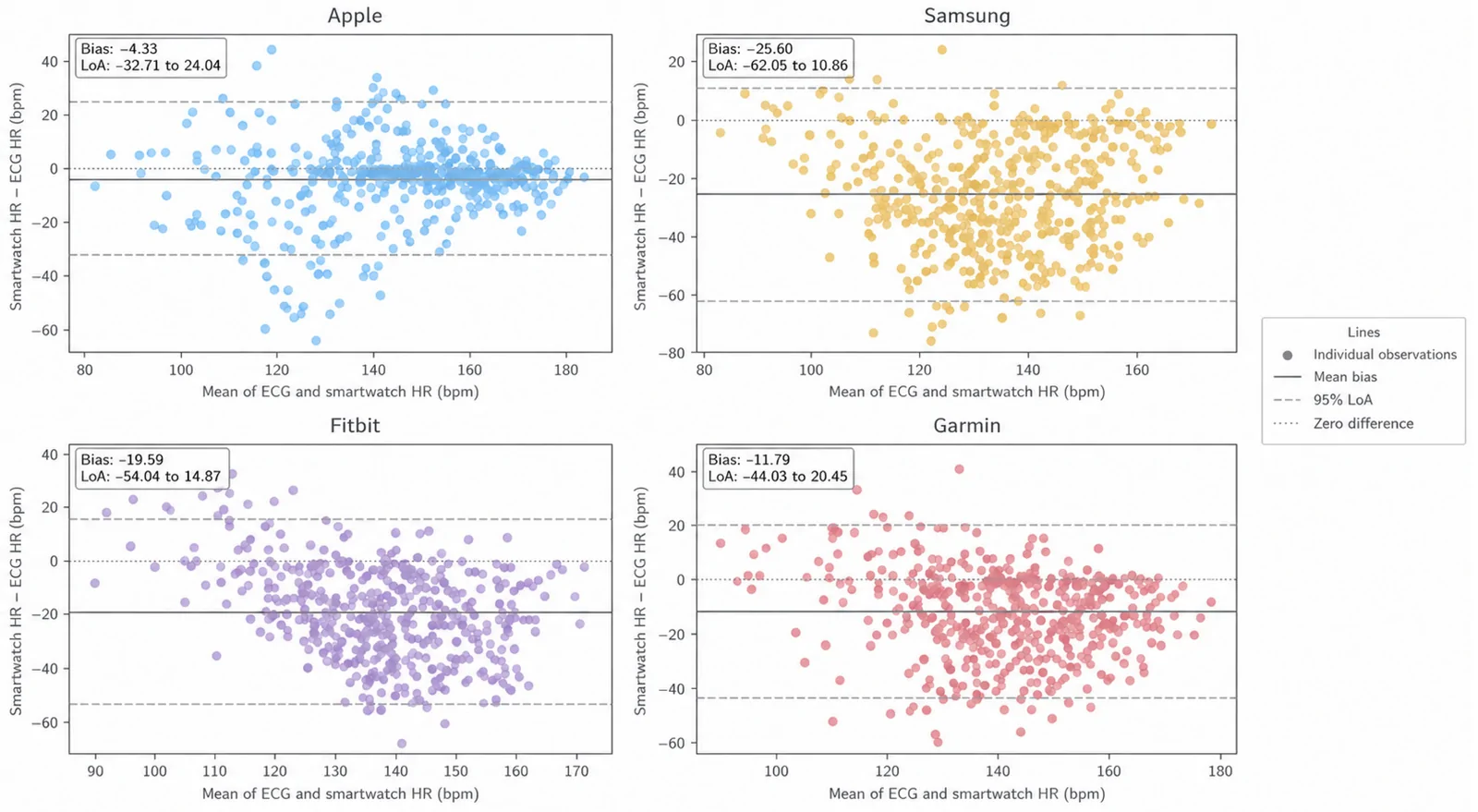
Bland–Altman plots of smartwatch-derived heart rate immediately post-set. The X-axis represents the mean of smartwatch-derived and ECG-derived heart rate, whereas the y-axis represents the difference between the two measurements, calculated as smartwatch-derived heart rate minus ECG-derived heart rate. Solid horizontal lines indicate the mean bias, dashed lines indicate the 95% LoA, and dotted lines indicate zero difference.

**Figure 5:**
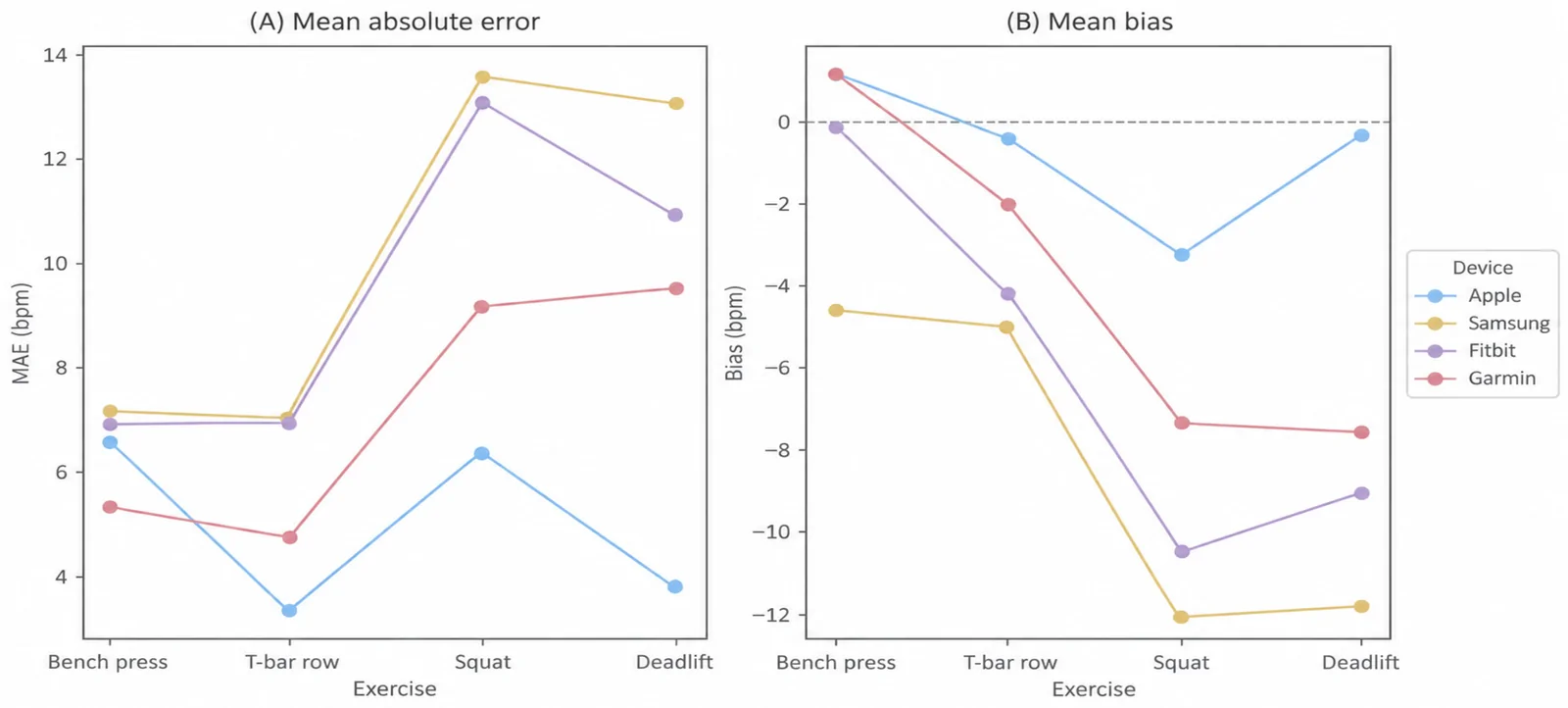
Exercise-context-specific errors in smartwatch-derived heart rate during the fixed resistance-exercise sequence. (**A**) MAE across the bench press, T-bar row, squat, and deadlift stages. (**B**) Mean bias, calculated as smartwatch-derived heart rate minus ECG-derived heart rate. Positive values indicate overestimation relative to ECG, whereas negative values indicate underestimation. Larger measurement errors occurred during the later squat and deadlift stages; however, because exercise type was completely confounded with protocol position, these differences should not be interpreted as isolated causal effects of exercise type. MAE = mean absolute error.

**Table 1:** Criterion validity of smartwatch-derived pre-protocol laboratory baseline heart rate, mean protocol heart rate, and energy expenditure.

Outcome	Device	N	Reference Mean ± SD	Smartwatch Mean ± SD	Bias [95% CI]	MAE	MAPE (%)	RMSE	ICC [95% CI]	95% LoA
Pre-protocol baseline HR	Apple	70	98.50 ± 14.63	97.60 ± 15.17	−0.90 [−1.89 to 0.04]	3.16	3.30	4.08	0.963 [0.94 to 0.98]	−8.76 to 6.96
Pre-protocol baseline HR	Samsung	70	98.50 ± 14.63	97.76 ± 15.85	−0.74 [−3.69 to 2.14]	7.37	7.19	12.64	0.655 [0.44 to 0.83]	−25.64 to 24.16
Pre-protocol baseline HR	Fitbit	70	98.50 ± 14.63	98.99 ± 13.49	0.49 [−1.23 to 2.53]	4.20	4.47	8.02	0.837 [0.64 to 0.95]	−15.32 to 16.29
Pre-protocol baseline HR	Garmin	70	98.50 ± 14.63	96.33 ± 14.70	−2.17 [−4.43 to −0.49]	4.60	4.51	8.66	0.827 [0.59 to 0.95]	−18.73 to 14.38
Mean protocol HR	Apple	70	125.37 ± 12.62	125.01 ± 12.29	−0.36 [−1.16 to 0.30]	1.81	1.44	3.08	0.969 [0.93 to 0.99]	−6.39 to 5.68
Mean protocol HR	Samsung	70	125.37 ± 12.62	121.01 ± 12.27	−4.36 [−5.27 to −3.60]	4.50	3.56	5.65	0.903 [0.84 to 0.94]	−11.45 to 2.74
Mean protocol HR	Fitbit	70	125.37 ± 12.62	121.97 ± 10.73	−3.40 [−4.36 to −2.50]	4.03	3.14	5.12	0.908 [0.86 to 0.94]	−10.97 to 4.17
Mean protocol HR	Garmin	70	125.37 ± 12.62	122.93 ± 12.12	−2.44 [−3.29 to −1.67]	3.10	2.44	4.28	0.941 [0.90 to 0.97]	−9.39 to 4.51
Energy expenditure	Apple	70	140.06 ± 14.23	296.30 ± 61.94	156.24 [142.91 to 170.04]	156.24	112.19	166.68	0.022 [0.01 to 0.04]	41.58 to 270.89
Energy expenditure	Samsung	70	140.06 ± 14.23	252.06 ± 60.60	112.00 [98.93 to 125.73]	117.25	84.48	126.08	0.029 [0.00 to 0.06]	−2.30 to 226.29
Energy expenditure	Fitbit	70	140.06 ± 14.23	147.34 ± 66.06	7.28 [−8.34 to 22.60]	56.77	41.73	66.39	0.033 [−0.06 to 0.14]	−122.98 to 137.55
Energy expenditure	Garmin	70	140.06 ± 14.23	300.03 ± 66.69	159.97 [145.85 to 174.55]	159.97	114.89	171.89	0.021 [0.01 to 0.04]	35.80 to 284.14

Note: Bias was calculated as the smartwatch-derived value minus the criterion value. Bias and ICCs are presented as point estimates with 95% confidence intervals. N indicates the number of paired observations. For pre-protocol baseline HR, mean protocol HR, and energy expenditure, each participant contributed one paired observation per device for each outcome; therefore, N = 70 also represents the number of participants. ECG served as the criterion method for heart rate, whereas indirect calorimetry served as the criterion method for energy expenditure. SD = standard deviation; CI = confidence interval; MAE = mean absolute error; MAPE = mean absolute percentage error; RMSE = root mean square error; ICC = intraclass correlation coefficient; LoA = limits of agreement; HR = heart rate.

**Table 2:** Overall validity of dynamic heart rate measurements during resistance exercise.

Device	N	ECG Mean ± SD	Smartwatch Mean ± SD	Bias [95% CI]	MAE	MAPE (%)	RMSE	ICC [95% CI]	Repeated Measures LoA
Apple	1680	129.95 ± 21.48	129.24 ± 20.83	−0.72 [−1.19 to −0.24]	4.95	3.79	9.28	0.904 [0.88 to 0.92]	−18.85 to 17.41
Samsung	1680	129.95 ± 21.48	121.52 ± 17.86	−8.44 [−9.01 to −7.84]	10.23	7.06	18.56	0.595 [0.55 to 0.63]	−40.86 to 23.98
Fitbit	1680	129.95 ± 21.48	123.97 ± 15.70	−5.98 [−6.60 to −5.36]	9.43	6.69	16.06	0.653 [0.62 to 0.69]	−35.21 to 23.24
Garmin	1680	129.95 ± 21.48	126.00 ± 18.12	−3.95 [−4.50 to −3.37]	7.18	5.17	12.82	0.796 [0.77 to 0.82]	−27.85 to 19.95

Note: Bias was calculated as smartwatch-derived heart rate minus ECG-derived heart rate. Bias and ICCs are presented as point estimates with 95% confidence intervals. N indicates the number of paired observations rather than the number of participants. Repeated-measures limits of agreement (LoA) account for within-participant dependence arising from repeated observations. SD = standard deviation; CI = confidence interval; MAE = mean absolute error; MAPE = mean absolute percentage error; RMSE = root mean square error; ICC = intraclass correlation coefficient; LoA = limits of agreement.

**Table 3:** Time point-specific validity of dynamic heart rate measurements during resistance exercise.

Time Point	Device	N	ECG Mean ± SD	Smartwatch Mean ± SD	Bias [95% CI]	MAE	MAPE (%)	RMSE	ICC [95% CI]	Repeated Measures LoA
Pre-set	Apple	560	114.73 ± 15.15	115.42 ± 14.65	0.68 [0.24 to 1.14]	3.02	2.76	4.39	0.957 [0.95 to 0.97]	−7.81 to 9.18
Pre-set	Samsung	560	114.73 ± 15.15	114.56 ± 15.26	−0.18 [−0.55 to 0.15]	2.42	2.19	4.08	0.964 [0.95 to 0.98]	−8.18 to 7.83
Pre-set	Fitbit	560	114.73 ± 15.15	116.07 ± 14.96	1.34 [0.65 to 2.11]	3.40	3.27	7.01	0.892 [0.82 to 0.94]	−12.16 to 14.85
Pre-set	Garmin	560	114.73 ± 15.15	114.80 ± 15.00	0.07 [−0.37 to 0.49]	2.86	2.62	4.99	0.945 [0.93 to 0.96]	−9.73 to 9.86
Immediately post-set	Apple	560	147.03 ± 18.98	142.70 ± 21.15	−4.33 [−5.64 to −3.06]	9.59	6.81	15.10	0.724 [0.65 to 0.79]	−32.71 to 24.04
Immediately post-set	Samsung	560	147.03 ± 18.98	121.44 ± 19.01	−25.60 [−27.20 to −24.01]	26.35	17.48	31.63	0.273 [0.23 to 0.32]	−62.05 to 10.86
Immediately post-set	Fitbit	560	147.03 ± 18.98	127.45 ± 13.33	−19.59 [−20.98 to −18.17]	21.96	14.51	26.31	0.248 [0.20 to 0.30]	−54.04 to 14.87
Immediately post-set	Garmin	560	147.03 ± 18.98	135.24 ± 16.40	−11.79 [−12.99 to −10.59]	15.38	10.33	20.23	0.467 [0.40 to 0.53]	−44.03 to 20.45
1 min post-set	Apple	560	128.09 ± 16.37	129.59 ± 16.37	1.50 [1.23 to 1.76]	2.26	1.81	3.30	0.980 [0.97 to 0.98]	−4.27 to 7.27
1 min post-set	Samsung	560	128.09 ± 16.37	128.56 ± 16.30	0.46 [0.06 to 0.83]	1.93	1.53	4.08	0.969 [0.94 to 0.99]	−7.48 to 8.41
1 min post-set	Fitbit	560	128.09 ± 16.37	128.39 ± 15.64	0.29 [−0.28 to 0.83]	2.93	2.30	5.73	0.936 [0.91 to 0.96]	−10.94 to 11.52
1 min post-set	Garmin	560	128.09 ± 16.37	127.97 ± 16.63	−0.12 [−0.98 to 0.59]	3.30	2.55	7.66	0.892 [0.85 to 0.93]	−15.15 to 14.90

Note: Bias was calculated as smartwatch-derived heart rate minus ECG-derived heart rate. Bias and ICCs are presented as point estimates with 95% confidence intervals. N indicates the number of paired observations rather than the number of participants. Repeated-measures limits of agreement (LoA) account for within-participant dependence arising from repeated observations. SD = standard deviation; CI = confidence interval; MAE = mean absolute error; MAPE = mean absolute percentage error; RMSE = root mean square error; ICC = intraclass correlation coefficient; LoA = limits of agreement.

**Table 4:** Exercise-specific validity of dynamic heart rate measurements during resistance exercise.

Exercise	Device	N	ECG Mean ± SD	Smartwatch Mean ± SD	Bias [95% CI]	MAE	MAPE (%)	RMSE	ICC [95% CI]	Repeated Measures LoA
Bench presses	Apple	420	117.34 ± 16.94	118.55 ± 19.02	1.21 [0.22 to 2.15]	6.48	5.48	10.22	0.839 [0.80 to 0.87]	−18.71 to 21.13
Bench presses	Samsung	420	117.34 ± 16.94	112.71 ± 15.44	−4.63 [−5.63 to −3.60]	7.10	5.71	11.95	0.738 [0.67 to 0.79]	−26.26 to 17.00
Bench presses	Fitbit	420	117.34 ± 16.94	117.25 ± 14.47	−0.09 [−1.38 to 1.15]	6.84	5.94	10.88	0.761 [0.71 to 0.81]	−21.45 to 21.26
Bench presses	Garmin	420	117.34 ± 16.94	118.55 ± 16.84	1.20 [0.23 to 2.25]	5.31	4.67	8.79	0.865 [0.81 to 0.90]	−15.88 to 18.29
T-bar rows	Apple	420	130.89 ± 19.07	130.48 ± 18.19	−0.41 [−1.01 to 0.16]	3.25	2.45	6.21	0.944 [0.92 to 0.97]	−12.58 to 11.76
T-bar rows	Samsung	420	130.89 ± 19.07	125.90 ± 17.05	−4.99 [−6.09 to −3.95]	7.03	4.95	13.59	0.728 [0.65 to 0.80]	−29.79 to 19.82
T-bar rows	Fitbit	420	130.89 ± 19.07	126.66 ± 15.60	−4.23 [−5.20 to −3.22]	6.92	4.94	12.51	0.750 [0.68 to 0.81]	−27.33 to 18.87
T-bar rows	Garmin	420	130.89 ± 19.07	128.89 ± 18.16	−2.00 [−3.23 to −0.91]	4.70	3.45	9.24	0.878 [0.81 to 0.93]	−19.71 to 15.70
Squats	Apple	420	133.96 ± 23.51	130.62 ± 22.00	−3.35 [−4.63 to −2.16]	6.31	4.49	12.76	0.845 [0.79 to 0.90]	−27.50 to 20.81
Squats	Samsung	420	133.96 ± 23.51	121.72 ± 18.93	−12.24 [−13.49 to −10.99]	13.67	8.95	24.08	0.453 [0.38 to 0.52]	−52.93 to 28.44
Squats	Fitbit	420	133.96 ± 23.51	123.48 ± 15.80	−10.48 [−11.82 to −9.17]	13.13	8.72	21.60	0.488 [0.42 to 0.55]	−47.55 to 26.59
Squats	Garmin	420	133.96 ± 23.51	126.59 ± 18.62	−7.38 [−8.54 to −6.20]	9.22	6.16	15.93	0.734 [0.69 to 0.78]	−35.07 to 20.32
Deadlifts	Apple	420	137.62 ± 20.24	137.30 ± 19.46	−0.32 [−0.95 to 0.30]	3.77	2.76	6.19	0.951 [0.94 to 0.96]	−12.44 to 11.80
Deadlifts	Samsung	420	137.62 ± 20.24	125.73 ± 16.61	−11.89 [−12.73 to −10.97]	13.12	8.66	21.71	0.430 [0.36 to 0.49]	−47.53 to 23.75
Deadlifts	Fitbit	420	137.62 ± 20.24	128.49 ± 14.56	−9.13 [−10.06 to −8.25]	10.83	7.16	17.05	0.588 [0.53 to 0.64]	−37.38 to 19.11
Deadlifts	Garmin	420	137.62 ± 20.24	130.00 ± 16.57	−7.62 [−8.83 to −6.47]	9.50	6.39	15.51	0.676 [0.62 to 0.73]	−34.14 to 18.89

Note: The exercises were performed in the following fixed order: bench press, T-bar row, squat, and deadlift. Bias was calculated as smartwatch-derived heart rate minus ECG-derived heart rate. Bias and ICCs are presented as point estimates with 95% confidence intervals. N indicates the number of paired observations rather than the number of participants. Repeated-measures limits of agreement (LoA) account for within-participant dependence arising from repeated observations. Because exercise type was completely confounded with protocol position, the exercise-specific results should not be interpreted as isolated causal effects of exercise type. SD = standard deviation; CI = confidence interval; MAE = mean absolute error; MAPE = mean absolute percentage error; RMSE = root mean square error; ICC = intraclass correlation coefficient; LoA = limits of agreement.

**Table 5:** Results of repeated-measures models for dynamic heart rate error and energy expenditure error.

Model	Effect	Wald χ^2^	df	*p*
Dynamic HR error repeated-measures model	Device	45.113	3	<0.001
Dynamic HR error repeated-measures model	Exercise	32.246	3	<0.001
Dynamic HR error repeated-measures model	Time point	97.08	2	<0.001
Dynamic HR error repeated-measures model	Set	0.0	1	0.985
Dynamic HR error repeated-measures model	Device × Time point	1011.868	6	<0.001
Dynamic HR error repeated-measures model	Device × Exercise	200.381	9	<0.001
Dynamic HR error repeated-measures model	Exercise × Time point	252.539	6	<0.001
EE error repeated-measures model	Device	854.084	3	<0.001

Note: For the dynamic HR analyses, the dependent variable was HR error, calculated as smartwatch-displayed heart rate minus ECG-displayed heart rate. For the EE analysis, the dependent variable was EE error, calculated as smartwatch-derived energy expenditure minus indirect calorimetry-derived energy expenditure. *p*-values < 0.001 are reported as <0.001. HR = heart rate; EE = energy expenditure.

## Data Availability

The data that support the findings of this study are available from the corresponding author upon reasonable request.

## Supplementary Materials

The following supporting information can be downloaded at: https://www.mdpi.com/article/10.3390/bioengineering13101099/s1, Table S1, Pairwise post hoc comparisons of overall dynamic heart rate error; Table S2, Pairwise post hoc comparisons of time point-specific dynamic heart rate error; Table S3, Pairwise post hoc comparisons of exercise-specific dynamic heart rate error; Table S4, Set-specific descriptive error estimates for dynamic heart rate by device; Table S5, Participant-clustered marginal mean heart rate error across exercise/protocol stage and time point; Table S6, Participant-level sensitivity analysis of overall dynamic heart rate agreement.
