# Supplementary material for: Criterion Validity of Smartwatches for Measuring Heart Rate and Energy Expenditure During Resistance Exercise
Source: Bioengineering (Basel). 2026 Sep 23;13(10):1099. doi: 10.3390/bioengineering13101099 (PMC13701894; doi:10.3390/bioengineering13101099)
Supplement: Supplementary file 1 [file bioengineering-13-01099-s001.zip › bioengineering-4575966-supplementary.pdf]

bioengineering

MDPI

Supplementary Materials

Criterion Validity of Smartwatches for Measuring Heart Rate and Energy Expenditure During Resistance Exercise

Jung-Hyun Yun [1,†], Jae-Myun Ko [2,†] and Tae-Hyung Lee [2,*]

1 Department of Sports Science, Konkuk University, 268 Chungwon-daero, Chungju-si, Chungcheongbuk-do 27478, Republic of Korea; 1221chyun@naver.com
2 Exercise Physiology Laboratory, Department of Physical Education, Yonsei University, Seoul 03722, Republic of Korea; kjm1218@yonsei.ac.kr
* Correspondence: th@yonsei.ac.kr (T.-H.L.); Tel.: +82-2-2123-3185; Fax: +82-2-2123-8375
† These authors contributed equally to this work.

Table S1. Pairwise post hoc comparisons of overall dynamic heart rate error.

Context	Comparison	n	Mean error difference	Test	Statistic	Raw p	Shapiro–Wilk p	Holm-adjusted p
Overall dynamic HR	Apple vs. Samsung	70	7.720	Paired t test	23.859	<0.001	0.734	<0.001
Overall dynamic HR	Apple vs. Fitbit	70	5.268	Paired t test	12.872	<0.001	0.121	<0.001
Overall dynamic HR	Apple vs. Garmin	70	3.233	Paired t test	9.501	<0.001	0.422	<0.001
Overall dynamic HR	Samsung vs. Fitbit	70	−2.452	Wilcoxon signed-rank test	289.000	<0.001	0.005	<0.001
Overall dynamic HR	Samsung vs. Garmin	70	−4.487	Wilcoxon signed-rank test	19.000	<0.001	0.003	<0.001
Overall dynamic HR	Fitbit vs. Garmin	70	−2.034	Wilcoxon signed-rank test	344.500	<0.001	<0.001	<0.001

Mean error difference represents the participant-level difference in mean heart rate error between the first and second devices listed in each comparison (first device minus second device). The Shapiro–Wilk test was applied to the paired differences to determine the choice of paired test. p values were adjusted for the six pairwise device comparisons using the Holm method. n = number of paired participants; HR = heart rate.

Table S2. Pairwise post hoc comparisons of time point-specific dynamic heart rate error.

Context	Comparison	n	Mean error difference	Test	Statistic	Raw p	Shapiro–Wilk p	Holm-adjusted p
Time point: Pre-set	Apple vs. Fitbit	70	−0.657	Wilcoxon signed-rank test	1021.000	0.353	<0.001	0.474
Time point: Pre-set	Apple vs. Garmin	70	0.618	Wilcoxon signed-rank test	657.000	0.002	0.028	0.006
Time point: Pre-set	Apple vs. Samsung	70	0.861	Paired t test	4.545	<0.001	0.269	<0.001
Time point: Pre-set	Fitbit vs. Garmin	70	1.275	Wilcoxon signed-rank test	611.500	0.003	<0.001	0.008
Time point: Pre-set	Samsung vs. Fitbit	70	−1.518	Wilcoxon signed-rank test	507.000	<0.001	<0.001	<0.001

Context	Comparison	n	Mean error difference	Test	Statistic	Raw p	Shapiro–Wilk p	Holm-adjusted p
Time point: Pre-set	Samsung vs. Garmin	70	−0.243	Paired t test	−1.193	0.237	0.080	0.474
Time point: Immediately post-set	Apple vs. Fitbit	70	15.254	Paired t test	15.918	<0.001	0.680	<0.001
Time point: Immediately post-set	Apple vs. Garmin	70	7.459	Paired t test	9.177	<0.001	0.938	<0.001
Time point: Immediately post-set	Apple vs. Samsung	70	21.266	Paired t test	24.641	<0.001	0.807	<0.001
Time point: Immediately post-set	Fitbit vs. Garmin	70	−7.795	Paired t test	−12.248	<0.001	0.857	<0.001
Time point: Immediately post-set	Samsung vs. Fitbit	70	−6.013	Paired t test	−7.395	<0.001	0.894	<0.001
Time point: Immediately post-set	Samsung vs. Garmin	70	−13.807	Paired t test	−23.944	<0.001	0.583	<0.001
Time point: 1-min post-set	Apple vs. Fitbit	70	1.207	Wilcoxon signed-rank test	429.000	<0.001	<0.001	<0.001
Time point: 1-min post-set	Apple vs. Garmin	70	1.623	Wilcoxon signed-rank test	451.000	<0.001	<0.001	<0.001
Time point: 1-min post-set	Apple vs. Samsung	70	1.034	Wilcoxon signed-rank test	388.000	<0.001	0.003	<0.001
Time point: 1-min post-set	Fitbit vs. Garmin	70	0.416	Wilcoxon signed-rank test	1219.000	0.891	0.002	1.000
Time point: 1-min post-set	Samsung vs. Fitbit	70	0.173	Wilcoxon signed-rank test	1038.000	0.528	<0.001	1.000
Time point: 1-min post-set	Samsung vs. Garmin	70	0.589	Wilcoxon signed-rank test	975.500	0.666	<0.001	1.000

Mean error difference represents the participant-level difference in mean heart rate error between the first and second devices listed in each comparison at each time point (first device minus second device). Time points were defined as follows: Pre-set indicates the measurement obtained immediately before a set; Immediately post-set indicates the measurement obtained within 2 s after completion of the 10th repetition; and 1-min post-set indicates the measurement obtained 1 min after completion of a set. The Shapiro–Wilk test was applied to the paired differences to determine the choice of paired test. Within each time point, p values were adjusted for the six pairwise device comparisons using the Holm method. n = number of paired participants; HR = heart rate.

https://doi.org/10.3390/bioengineering13101099

Table S3. Pairwise post hoc comparisons of exercise-specific dynamic heart rate error.

Context	Comparison	n	Mean error difference	Test	Statistic	Raw p	Shapiro–Wilk p	Holm-adjusted p
Bench press	Apple vs. Fitbit	70	1.305	Wilcoxon signed-rank test	789.000	0.029	0.016	0.058
Bench press	Apple vs. Garmin	70	0.007	Paired t test	0.011	0.991	0.196	0.991
Bench press	Apple vs. Samsung	70	5.840	Wilcoxon signed-rank test	35.000	<0.001	0.003	<0.001
Bench press	Fitbit vs. Garmin	70	−1.298	Wilcoxon signed-rank test	775.500	0.006	0.003	0.019
Bench press	Samsung vs. Fitbit	70	−4.536	Wilcoxon signed-rank test	205.000	<0.001	0.011	<0.001
Bench press	Samsung vs. Garmin	70	−5.833	Paired t test	−12.862	<0.001	0.141	<0.001
T-bar row	Apple vs. Fitbit	70	3.819	Paired t test	7.317	<0.001	0.506	<0.001
T-bar row	Apple vs. Garmin	70	1.591	Wilcoxon signed-rank test	863.500	0.059	<0.001	0.117
T-bar row	Apple vs. Samsung	70	4.576	Wilcoxon signed-rank test	44.000	<0.001	<0.001	<0.001
T-bar row	Fitbit vs. Garmin	70	−2.229	Wilcoxon signed-rank test	379.000	<0.001	<0.001	<0.001
T-bar row	Samsung vs. Fitbit	70	−0.757	Wilcoxon signed-rank test	1132.500	0.520	0.012	0.520
T-bar row	Samsung vs. Garmin	70	−2.986	Wilcoxon signed-rank test	283.500	<0.001	0.002	<0.001
Squat	Apple vs. Fitbit	70	7.133	Wilcoxon signed-rank test	206.000	<0.001	0.007	<0.001
Squat	Apple vs. Garmin	70	4.031	Paired t test	6.102	<0.001	0.564	<0.001
Squat	Apple vs. Samsung	70	8.895	Wilcoxon signed-rank test	13.000	<0.001	0.036	<0.001
Squat	Fitbit vs. Garmin	70	−3.102	Paired t test	−4.317	<0.001	0.057	<0.001
Squat	Samsung vs. Fitbit	70	−1.762	Wilcoxon signed-rank test	837.500	0.040	0.033	0.040
Squat	Samsung vs. Garmin	70	−4.864	Wilcoxon signed-rank test	215.500	<0.001	0.006	<0.001
Deadlift	Apple vs. Fitbit	70	8.814	Paired t test	15.994	<0.001	0.625	<0.001
Deadlift	Apple vs. Garmin	70	7.305	Wilcoxon signed-rank test	32.000	<0.001	0.001	<0.001
Deadlift	Apple vs. Samsung	70	11.569	Wilcoxon signed-rank test	1.000	<0.001	0.030	<0.001
Deadlift	Fitbit vs. Garmin	70	−1.510	Wilcoxon signed-rank test	705.000	0.003	0.040	0.003
Deadlift	Samsung vs. Fitbit	70	−2.755	Wilcoxon signed-rank test	400.000	<0.001	0.030	<0.001
Deadlift	Samsung vs. Garmin	70	−4.264	Wilcoxon signed-rank test	232.000	<0.001	0.036	<0.001

Mean error difference represents the participant-level difference in mean heart rate error between the first and second devices listed in each comparison for each exercise/protocol stage (first device minus second device). The Shapiro–Wilk test was applied to the paired differences to determine the

choice of paired test. Within each exercise/protocol stage, p values were adjusted for the six pairwise device comparisons using the Holm method. The exercises were performed in the following fixed order: bench press, T-bar row, squat, and deadlift. Because exercise type was completely confounded with protocol position, these exercise-specific comparisons should not be interpreted as isolated causal effects of exercise type. n = number of paired participants; HR = heart rate.

Table S4. Set-specific descriptive error estimates for dynamic heart rate by device.

Device	Set	N	Bias [95% CI] (bpm)	MAE (bpm)	RMSE (bpm)
Apple	Set 1	840	−0.96 [−1.51 to −0.40]	5.26	9.68
Apple	Set 2	840	−0.47 [−1.07 to 0.12]	4.64	8.85
Samsung	Set 1	840	−8.51 [−9.21 to −7.79]	10.48	18.82
Samsung	Set 2	840	−8.36 [−8.97 to −7.73]	9.99	18.30
Fitbit	Set 1	840	−5.45 [−6.26 to −4.54]	9.51	15.96
Fitbit	Set 2	840	−6.52 [−7.14 to −5.92]	9.35	16.17
Garmin	Set 1	840	−4.18 [−4.98 to −3.37]	7.96	13.99
Garmin	Set 2	840	−3.73 [−4.25 to −3.19]	6.41	11.52

Bias was calculated as smartwatch-derived HR minus ECG-derived HR; positive values indicate smartwatch overestimation, whereas negative values indicate smartwatch underestimation relative to ECG. The 95% confidence intervals for bias were estimated using participant-clustered bootstrap resampling. N denotes the number of paired observations; each participant contributed 12 observations per set for each device (four exercises × three time points), yielding N = 840 per device and set. The repeated-measures model showed no statistically significant main effect of set (p = .985). CI = confidence interval; MAE = mean absolute error; RMSE = root mean square error; HR = heart rate.

Table S5. Participant-clustered marginal mean heart rate error across exercise/protocol stage and time point.

Exercise/protocol stage	Time point	Mean HR error [95% CI] (bpm)
Bench press	Pre-set	1.08 [0.30 to 1.86]
Bench press	Immediately post-set	−3.57 [−5.68 to −1.42]
Bench press	1-min post-set	0.77 [0.35 to 1.22]
T-bar row	Pre-set	0.71 [0.24 to 1.20]
T-bar row	Immediately post-set	−10.42 [−12.34 to −8.50]
T-bar row	1-min post-set	0.99 [0.42 to 1.43]
Squat	Pre-set	0.49 [−0.03 to 0.98]
Squat	Immediately post-set	−26.06 [−28.34 to −23.76]
Squat	1-min post-set	0.49 [−0.08 to 0.99]
Deadlift	Pre-set	−0.36 [−0.92 to 0.24]
Deadlift	Immediately post-set	−21.25 [−23.12 to −19.39]
Deadlift	1-min post-set	−0.11 [−0.87 to 0.61]

Values are participant-clustered marginal mean HR errors across the four devices, with 95% confidence intervals estimated using participant-clustered bootstrap resampling. HR error was calculated as smartwatch-displayed HR minus ECG-displayed HR; positive values indicate smartwatch overestimation, whereas negative values indicate smartwatch underestimation relative to ECG. The exercise × time point interaction in the sensitivity analysis was statistically significant (Wald $\chi^2(6)$ = 252.54, p < .001), indicating that the temporal pattern of HR error varied across the fixed exercise/protocol stages. Because exercise order was fixed (bench press → T-bar row → squat → deadlift), exercise type was completely confounded with protocol position and accumulated prior workload; therefore, these stage-specific estimates should not be interpreted as isolated causal effects of exercise type. CI = confidence interval; HR = heart rate.

Table S6. Participant-level sensitivity analysis of overall dynamic heart rate agreement.

Device	Participants	Participant-level ICC [95% CI]
Apple	70	0.963 [0.939 to 0.976]
Samsung	70	0.599 [0.506 to 0.667]
Fitbit	70	0.713 [0.628 to 0.773]
Garmin	70	0.841 [0.780 to 0.884]

For this sensitivity analysis, each participant’s 24 dynamic HR observations per device (four exercises × two sets × three time points) were averaged, and the two-way mixed-effects, absolute-agreement, single-measure ICC was recalculated using the 70 participant-level paired means. The 95% confidence intervals were estimated using participant-level bootstrap resampling. This analysis complements the primary measurement-occasion-level ICC analysis by treating the participant, rather than the individual measurement occasion, as the statistical target. CI = confidence interval; HR = heart rate; ICC = intraclass correlation coefficient.

https://doi.org/10.3390/bioengineering13101099
